# Transcriptomic analysis reveals the landscape of the shared gene network between ectopic pregnancy and early pregnancy loss

**DOI:** 10.1016/j.gendis.2025.101616

**Published:** 2025-03-27

**Authors:** Mengyu Jing, Ying Zhou, Shuyue Zheng, Yahui Xie, Xi Chen, Aixia Liu

**Affiliations:** a Department of Reproductive Endocrinology, Women’s Hospital, Zhejiang University School of Medicine, Hangzhou, Zhejiang 310006, China; b Key Laboratory of Reproductive Genetics (Ministry of Education), Zhejiang University, Hangzhou, Zhejiang 310006, China; c Zhejiang Key Laboratory of Maternal and Infant Health, Zhejiang University, Hangzhou, Zhejiang 310006, China; d Zhejiang Provincial Clinical Research Center for Child Health, Women’s Hospital, Zhejiang University School of Medicine, Hangzhou, Zhejiang 310006, China

The loss of a pregnancy, including ectopic pregnancy (EP) and early pregnancy loss (EPL), significantly impacts women’s quality of life. Unfortunately, definitive causes can be identified in less than half of EP and EPL cases, presenting a substantial challenge for clinical treatment. Previous studies have revealed a significant relationship between the history of EPL and the increased risk of EP.^1^ Nevertheless, the interplay between EPL and EP remains unclear, highlighting the need to discover novel biomarkers to guide personalized treatment and clinical management. In response, we aim to investigate the underlying genetic interactions in EPL and EP. Using various bioinformatics analyses, we examined the genetic interactions of differentially expressed genes (DEGs) through RNA sequencing according to the workflow (Fig. S1).

Villous tissues were collected from patients with EPL, EP, and those undergoing unwanted pregnancies (referred to as the normal pregnancy group). A total of 36 patients participated in this study,with 15 used for RNA sequencing and 21 for validation. Detailed information is provided in Table S1. All patients signed informed consent forms. The quality control results showed a high quality of raw data for each sample, which could be utilized for subsequent analyses (Table S2). With the cut-off criteria of |log_2_fold change| > 1 and false discovery rate (FDR) < 0.05 upon DESeq2 package, 1220 DEGs could be observed in EPL patients, compared with 640 DEGs in EP patients. Ultimately, 241 shared DEGs were identified between the EPL and EP groups (Fig. 1A; Figs. S2A and B). Interestingly, 233 of these shared DEGs exhibited the same expression trend, suggesting a high degree of similarity in the underlying molecular mechanisms between EPL and EP. The biological functions and potential pathways were evaluated using gene ontology (GO) and Kyoto Encyclopedia of Genes and Genomes (KEGG) pathway analysis through the DAVID database (https://david.ncifcrf.gov/). The results showed significant enrichment in multiple immune- and inflammation-related pathways, such as the Toll-like receptor signaling pathway (FDR <0.05) (Fig. 1B; Figs. S2C–E).

**Figure 1:**
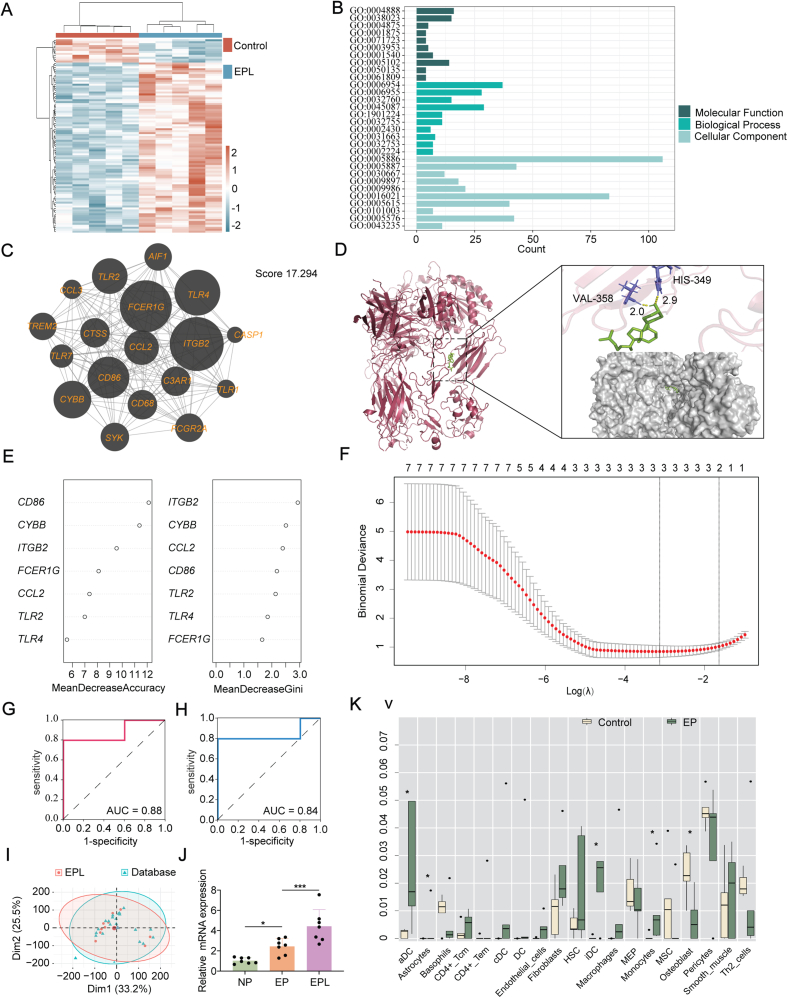
The landscape of the shared gene network between early pregnancy loss (EPL) and ectopic pregnancy (EP). **(A)** The heatmap of differentially expressed genes (DEGs) in EPL patients. **(B)** The bar chart of GO functional analysis for shared DEGs. **(C)** The core module identified in the protein–protein interaction network. **(D)** Molecular docking schematic of cholecalciferol and *ITGB2*. **(E)** Machine learning of random forest analysis. **(F)** Machine learning of LASSO analysis. **(G, H)** Diagnostic potential of the *CYBB* gene in EPL (G) and EP (H) patients. **(I)** Principal component analysis plots after batch correction with dataset GSE123719. **(J)** The relative expression levels in *CYBB* were validated by quantitative reverse transcription PCR on a sample set of seven EPL patients, seven EP patients, and seven controls. **(K)** Comparison of immune cell fractions between EP patients and controls (extremely low levels of immune cells were excluded). ∗*p* < 0.05 and ∗∗∗*p* < 0.001.

Next, a protein–protein interaction network was constructed through the STRING database (http://string-db.org/) (Fig. S3A). Comprising 18 shared DEGs, we identified a core module with a score of 17.294 by MCODE in Cytoscape 3.8.2 (Fig. 1C). Functional analysis revealed significant enrichment of various immune-inflammatory pathways in the core module (Figs. S3B and C). Simultaneously, we applied 12 algorithms under CytoHubba to identify the top 10 hub DEGs (Table S3). Based on their frequencies, the top 10 DEGs with the highest frequencies were defined as hub DEGs (*PTPRC*, *TLR4*, *TYROBP*, *CCL2*, *ITGB2*, *FCER1G*, *LCP2*, *CD86*, *CYBB*, *TLR2*). These hub DEGs were intersected with the core module, resulting in seven hub-shared DEGs (Fig. S4A). Next, we established a co-expression network using GeneMANIA (http://genemania.org/) to investigate the associated interactive genes. Functional analysis revealed this co-expression network, consisting of 7 hub-shared DEGs and 20 co-expressed genes, was closely associated with immune-inflammatory responses, such as the migration and activation of immune cells (FDR <0.05; Fig. S4B). Based on these findings, we hypothesized that an immune-inflammatory response may be one of the common underlying factors in EPL and EP patients.

Assessing the interactions between proteins and drugs constitutes is a critical step in the drug discovery and development process. In this study, 135 small-molecule drugs were predicted (adjusted *p* < 0.05) by the Drug Signatures Database (https://dsigdb.tanlab.org/DSigDBv1.0/). Cholecalciferol, commonly known as vitamin D3, emerged as the most promising drug based on its combined score (Table S4). To further explore the impact of cholecalciferol on target genes and their drugability, we performed molecular docking of cholecalciferol with the targetable hub-shared DEGs, including *ITGB2*, *TLR4*, and *TLR2* (Fig. 1D; Figs. S4C and D) using Autodock 4.0 (http://autodock.scripps.edu/). Cholecalciferol and Protein Data Bank IDs utilized in this study are provided in Table S5 for reference. Our study suggests that cholecalciferol, considered safe for pregnant and lactating individuals,^2^ may possess preventive properties in those experiencing EPL/EP. Notably, for patients with a history of adverse pregnancy outcomes, such as EPL or EP, supplementing with cholecalciferol during subsequent pregnancies may help improve the chances of a successful outcome. Further investigation is needed to determine the optimal dosage of vitamin D supplements and to assess the specific benefits experienced by patients.

To identify feature DEGs, we first merged and batch-corrected the sequencing expression values of EPL and normal pregnancy patients with the GSE123719 dataset from the Gene Expression Omnibus database (https://www.ncbi.nlm.nih.gov/geo/) (Fig. 1I; Fig. S5A). Based on the filtering criterion of MeanDecreaseGini >2.5, we ultimately identified two feature hub-shared DEGs (*CYBB* and *ITGB2*) through random forest analysis with ntree set to 500 (Fig. 1E; Fig. S5B). We then employed LASSO regression and random forest analysis. Three feature hub-shared DEGs (*CD86*, *CYBB*, and *FCER1G*) were identified with the most appropriate lambda.min of 0.044 by LASSO analysis (Fig. 1F; Fig. S5C). Using both algorithms, we ultimately obtained *CYBB* as the signature hub-shared DEGs (Fig. S5D). The receiver operating characteristic curves demonstrated excellent diagnostic performance of *CYBB* in identifying EPL and EP (area under the curve > 0.7; Fig. 1G, H). *CYBB*, also known as *NOX2*, encodes the central protein of the NADPH oxidase multimeric complex,^3^ was found to be significantly upregulated in EPL/EP patients based on sequencing results. The up-regulated expression levels of *CYBB* were further confirmed through quantitative reverse transcription PCR (Table S6 and Fig. 1J). The abnormal expression of *CYBB* may contribute to the impaired ability of trophoblast cells to sense and respond to adverse embryonic growth conditions, potentially affecting pregnancy outcomes. Nevertheless, the exact cause of the increase of *CYBB* is still under investigation, considering its crucial role in oxidative stress and immune response.

*CYBB*, a key regulator of reactive oxygen species production, particularly in neutrophils and macrophages, is closely associated with oxidative stress.^4^ Analysis of immune infiltration through the xCell algorithm demonstrated a strongly positive correlation between *CYBB* and the levels of activated dendritic cells, immature dendritic cells, and macrophages in both EPL and EP (*p* < 0.05; Fig. S6A). Additionally, significant changes in immune cell infiltration were observed in the EPL and EP group (Fig. 1K; Fig. S6B), highlighting broader immune alterations. Consistently, a single-cell atlas of *CYBB* in placental tissues from the Human Protein Atlas database (https://www.proteinatlas.org/) similarly revealed its high expression with Hofbauer cells, which are a specific type of fetal-derived macrophages (Fig. S6C).

Effective management of EPL and EP is clinically crucial due to their significant impact on maternal health, as emphasized in the NICE guidelines, which highlight the importance of timely diagnosis and appropriate intervention.^5^ Notably, the *CYBB* gene emerged as a hub-shared DEG, suggesting potential preventive and therapeutic implications. However, further studies are needed to clarify its exact mechanisms and clinical applications.

## CRediT authorship contribution statement

**Mengyu Jing:** Data curation, Formal analysis, Writing – original draft, Writing – review & editing, Visualization. **Ying Zhou:** Resources. **Shuyue Zheng:** Visualization. **Yahui Xie:** Investigation. **Xi Chen:** Validation. **Aixia Liu:** Conceptualization, Funding acquisition, Supervision, Writing – review & editing.

## Ethics declaration

This study was approved by the Ethical Review Committee of Women’s Hospital, School of Medicine, Zhejiang University (approval number: 20180192). All patients included in this study signed informed consent forms.

## Funding

This research was supported by the Hangzhou Joint Fund of the 10.13039/501100004731Zhejiang Provincial Natural Science Foundation of China (No. LHZSD24H290001).

## Conflict of interests

The authors declared no competing interests.

## References

[1] Ticconi C., Capogna M.V., Martelli F. (2018). Ectopic pregnancy in women with recurrent miscarriage. J Obstet Gynaecol Res.

[2] García Martín A., Alhambra Expósito M.R., Cortés Berdonces M. (2022). Guide of management of alterations in mineral and bone metabolism during gestation and lactation. Endocrinol Diabetes Nutr (Engl Ed).

[3] Keller C.W., Kotur M.B., Mundt S. (2021). CYBB/NOX2 in conventional DCs controls T cell encephalitogenicity during neuroinflammation. Autophagy.

[4] Begum R., Thota S., Abdulkadir A., Kaur G., Bagam P., Batra S. (2022). NADPH oxidase family proteins: signaling dynamics to disease management. Cell Mol Immunol.

[5] Webster K., Eadon H., Fishburn S., Kumar G., Committee G. (2019). Ectopic pregnancy and miscarriage: diagnosis and initial management: summary of updated NICE guidance. BMJ.

