# Supplementary material for: Transcriptomic analysis reveals the landscape of the shared gene network between ectopic pregnancy and early pregnancy loss
Source: Genes Dis. 2025 Mar 27;12(6):101616. doi: 10.1016/j.gendis.2025.101616 (PMC12275975; doi:10.1016/j.gendis.2025.101616)
Supplement: Multimedia component 1 [file mmc1.docx]

**Transcriptomic Analysis Reveals the Landscape of the Shared Gene Network between Ectopic Pregnancy and Early Pregnancy Loss**

Mengyu Jing^a,b,c,d1^, Ying Zhou^a,b,c,d1^, Shuyue Zheng^a,b,c,d^, Yahui Xie^a,b,c,d^, Xi Chen^a,b,c,d^, Aixia Liu^a,b,c,d*^

^a^Department of Reproductive Endocrinology, Women’s Hospital, Zhejiang University School of Medicine, 1 Xueshi Road, Hangzhou 310006, P.R. China.

^b^Key Laboratory of Reproductive Genetics (Ministry of Education), Zhejiang University, Hangzhou 310006, P.R. China.

^c^Zhejiang Key Laboratory of Maternal and Infant Health, Zhejiang University, Hangzhou 310006, P.R. China.

^d^Zhejiang Provincial Clinical Research Center for Child Health, Women’s Hospital, Zhejiang University School of Medicine, Hangzhou, Zhejiang, 310006, P.R. China.

^*^Corresponding Author: Aixia Liu, liuaixia@zju.edu.cn.

**Additional Introduction**

The loss of a pregnancy, including ectopic pregnancy (EP) and early pregnancy loss (EPL), can have a negative impact on the quality of life of many women. Globally, approximately 23 million pregnancies result in miscarriage each year, an average of 44 per minute, with 80% occurring in the early stages ^1,2,3^. According to the American College of Obstetricians and Gynecologists (ACOG) guidelines, early pregnancy loss is defined as a nonviable pregnancy occurring before 12 6/7 weeks ^4^. Ectopic pregnancy refers to the implantation of the gestational sac outside the uterine cavity, with an incidence of approximately 1% ^5^. Tubal EP, in which an embryo implants in the fallopian tube, is the most common subtype ^6^.

EPL and EP share several common characteristics: they tend to exhibit repetitiveness in the same patient^7,8^, and increasing incidence in older women^9,10^. In a large-scale case-control population analysis conducted in France between 1993 and 2000, a close association was found between miscarriage and ectopic pregnancy, which was characterized by a "dose-related" response ^11^. Carlo Ticconi et al. ^12^ also identified an increased risk of EP in patients who have a miscarriage in their reproductive history. These findings have revealed a substantial correlation between EPL and EP.

Unfortunately, for both EP and EPL, a definitive cause can only be identified in less than half of cases, which poses a major challenge in clinical treatment ^13,14^. The incidence of pregnancy loss has been on the rise these years, influenced by factors such as delayed childbearing age and lifestyle changes ^15^. Recent research has concentrated on genetic and epigenetic polymorphisms mainly associated with immune response and inflammatory pathways^16,17^. Parameters linked to inflammation, and angiogenesis in the immune system may play an important but incompletely understood role in EP^18^. Prior research has elucidated a robust correlation between the extent of tubal dysfunction and the susceptibility to EP^19,20^. Despite these findings, the quest for a validated biomarker delineating the risk of EP in individuals with a history of EPL remains unfulfilled. The discovery of a novel, cost-effective biomarker for cases of EPL/EP holds promise in guiding treatment strategies and optimizing patient care, offering clinicians valuable tools for personalized intervention and improved management of affected individuals.

Recent rapid advances in RNA sequencing (RNA-Seq) provide researchers with remarkably large datasets and open new perspectives in the landscape of gene expression profiles. To explore the pathological mechanisms of EPL and EP and to assess the diagnostic and therapeutic potential of differentially expressed genes (DEGs) in chorionic villi, we conducted RNA-Seq analysis to investigate the potential genetic interactions among DEGs.

**Materials and methods**

**Clinical Patient Information**

We collected villous tissues of patients who attended the gynecology outpatient clinic of Women’s Hospital School of Medicine Zhejiang University for EPL, EP, and unwanted pregnancies (Approval number, 20180192). The diagnostic criteria for EPL were established in accordance with the guidelines provided by ACOG^4^. Choronic villi were collected from women undergoing laparoscope of tubal EP based on the criteria by the Royal College of Obstetricians and Gynaecologists and Association of Early Pregnancy Units^21^. All patients have signed informed consent forms.

**RNA-seq**

Chorionic villi were utilized for total RNA extraction employing the Magzol Reagent (Magen, China) as per the manufacturer's instructions. The quantity and integrity of the RNA obtained were evaluated using the K5500 (BeijingKaiao, China) and the Agilent 2200 TapeStation (Agilent Technologies, USA) individually. To eliminate rRNAs, the Total RNA was treated with the QIAseqFastSelct-rRNA HRM KIT (QIAGEN, Germany) and then fragmented to approximately 200bp. Subsequently, the purified RNA fragments underwent first-strand and second-strand cDNA synthesis, followed by adaptor ligation and enrichment with a low cycle, as per the instructions of the NEBNext® Ultra™ Directional RNA Library Prep Kit for Illumina (NEB, USA). The quality of the purified library products was assessed using the Agilent 2200 TapeStation and Qubit (Thermo Fisher Scientific, USA). The libraries were sequenced with paired-end 150bp on an Illumina platform (Illumina, USA) at Ribobio Co. Ltd (Ribobio, China).

**Identification and Functional Analysis of shared DEGs**

Expression analysis of DEGs was carried out employing the DESeq2 package, a widely used tool for RNA-Seq data analysis, with read counts as the input. DESeq2 analysis facilitates the identification of DEGs while controlling for false discovery rate (FDR), thereby enhancing the reliability and reproducibility of results. Genes with significant differential expression were identified based on stringent criteria, including a fold change > 2 and FDR < 0.05.

Functional analysis of DEGs involves various approaches to elucidate their biological significance. In this study, Gene Ontology (GO) and Kyoto Encyclopedia of Genes and Genomes (KEGG) pathway enrichment analyses were conducted using the DAVID database (https://david.ncifcrf.gov/). The primary objective of these analyses was to pinpoint enriched terms within GO categories, as well as KEGG pathways that are closely linked with the DEGs identified. By scrutinizing the shared DEGs exhibiting significant enrichment within these GO and KEGG terms, we aimed to unveil pivotal biological processes and molecular pathways underlying the observed gene expression alterations. The shared DEGs that significantly enriched these GO and KEGG terms were identified based on adjusted P values below 0.05.

**Construction and Analysis of Protein-Protein Interaction Networks and Modules**

The Search Tool for the Retrieval of Interacting Genes (STRING, http://string-db.org/, version 12.0) serves as a powerful platform for delving into the intricate connections among proteins of interest. The construction of a protein-protein interaction (PPI) network was facilitated by utilizing the STRING database, a comprehensive resource renowned for its extensive coverage of protein interactions across diverse biological contexts. Interactions scoring above 0.4 on the combined score scale were considered to be statistically significant, reflecting robust evidence of potential functional associations between proteins. The PPI network was visualized utilizing Cytoscape (version 3.8.2), a powerful bioinformatics software. The Cytoscape plugin MCODE was utilized to pinpoint crucial functional modules within the PPI network. Specific criteria were established for module identification, including a K-core of 2, a degree cutoff of 2, a maximum depth of 100, and a node score cutoff of 0.2.

**Identification and Functional Analysis of Hub-shared DEGs**

Identification of hub genes was carried out using the cytoHubba application within Cytoscape (version 3.8.2), which is a powerful tool designed for the analysis of biological networks, particularly in the identification of highly connected nodes or hub genes. We employed 12 algorithms (MCC, MNC, EPC, Stress, DMNC, Degree, Betweenness, BottleNeck, ClusteringCoefficient, EcCentricity, Closeness, and Radiality) to evaluate hub-shared DEGs and selected the top 10 genes from each. The TOP 10 genes obtained from each algorithm were sorted based on their frequencies, and the top 10 genes with the highest frequencies were defined as hub genes. GeneMANIA (http://genemania.org/) is a versatile online platform designed for predicting protein-protein interactions by leveraging gene functions. In this study, we utilized GeneMANIA to build a network of interactive proteins associated with hub genes ^22^.

**Candidate drug prediction and Molecular docking**

Assessing interactions between proteins and drugs constitutes a pivotal phase in the drug discovery and development process, pivotal for pinpointing promising drug targets and comprehending the therapeutic ramifications associated with target proteins. The shared DEGs uncovered in our investigation were submitted to the Drug Signatures Database (DSigDB) for comprehensive analysis. DSigDB, available at http://dsigdb.tanlab.org/DSigDBv1.0/, offers a robust platform for exploring the associations between identified DEGs and known drug signatures. The results generated by DSigDB provide insights into the therapeutic relevance of the shared DEGs and facilitate the identification of promising drug candidates for further investigation.

To deepen our comprehension of the impact of drug candidates on target genes and their drugability, this study delved into molecular docking at the atomic level. This allowed for the evaluation of binding energy and interaction patterns between drug candidates and their respective targets. Autodock 4.0 (http://autodock.scripps.edu/), a sophisticated protein-ligand docking software, was employed to conduct molecular docking analyses of the identified drug candidates with the proteins encoded by their corresponding target genes. This computational approach facilitates the exploration of binding interactions between drugs and their target proteins at the atomic level, allowing for the assessment of binding affinities and interaction patterns. Drug structure data were sourced from the PubChem Compound Database (https://pubchem.ncbi.nlm.nih.gov/), where each drug was assigned a unique identifier. Protein structure data were obtained from the Protein Data Bank (PDB), accessible at http://www.rcsb.org/. Each protein was allocated a distinct PDB identifier, facilitating the retrieval and analysis of its structural information. The corresponding drugs and PDB IDs utilized in this study are provided in Table S2 for reference.

**Dataset Merging and Machine Learning**

The National Center for Biotechnology Information (NCBI, https://www.ncbi.nlm.nih.gov/) is a vital bioinformatics resource center committed to providing data resources, tools, and services in the fields of biomedical research and genomics to scientists and researchers. We utilized the important global genome sequence database within NCBI, the GEO database, to acquire GSE123719, a transcriptome sequencing dataset for chorionic villi tissue from both early pregnancy loss and normal control groups. Batch effects are common issues in high-throughput data analysis. To mitigate the impact of batch effects on analysis results by adjusting batch effects within the data, this study applies the Combat algorithm. The Combat algorithm has been widely used in the analysis of gene expression data and other high-throughput data, providing researchers with an effective tool to correct batch effects and reduce data variability^23^.

The LASSO analysis was performed with parameters set as alpha = 1, indicating the L1 regularization penalty, and lambda_min was selected as the optimal lambda value. This approach allowed for the identification of the most relevant predictors among the variables considered, facilitating the construction of a parsimonious and predictive regression model. The random forest analysis was conducted utilizing the "randomForest" function in R. For determining the optimal parameters, the minimum error was selected as the mtry node value, ensuring robust variable selection. Additionally, the ntree value, representing the number of trees in the forest, was set to a stable value based on convergence criteria. Subsequently, a random forest tree model was constructed, leveraging these established parameters to facilitate accurate prediction and classification tasks. Two metrics, "Mean Decrease Accuracy" and "Mean Decrease Gini," are employed within the constructed random forest model to measure variable contribution. Specifically, "Mean Decrease Accuracy" signifies the reduction in prediction accuracy in a random forest model, while "Mean Decrease Gini" evaluates the contribution of each variable to the diversity of observed values at every node of the classification tree, thereby enabling a comparative analysis of variable importance ^24^.

**Validation of Gene Expression by Reverse Transcription Polymerase Chain Reaction**

In order to validate the expression levels of core feature genes in villous tissues, we collected chorionic villi from 7 EPL patients,7 EP patients, and 7 NP patients. The reverse transcription quantitative polymerase chain reaction (RT-qPCR) was employed to evaluate the expression levels of hub feature genes identified in the study.Total RNA extracted from chorionic villi was reverse transcribed into cDNA using the PrimeScript RT Reagent Kit with gDNA Eraser (Takara Bio Inc, Shiga, Japan). The validation of hub gene differential expression was conducted through RT-qPCR (Quantagene q900, China) using SYBR Premix Ex Taq II (Takara Bio Inc, Shiga, Japan). The program is configured as follows: 1) Pre-denaturation at 95°C for 30 seconds, 2) PCR reaction at 95°C for 5 seconds, 60°C for 30 seconds, repeated for 40 cycles, 3) Melting curve analysis. To ensure the accuracy of the results, each experimental group was subjected to three technical replicates. The primer sequences for RNA are provided in Table S3. Gene expression levels were normalized relative to β-actin and subsequently analyzed employing the threshold cycle (Ct) method. This standardization process ensures consistency and accuracy in quantifying gene expression across samples, allowing for precise comparisons and reliable interpretation of experimental results.

**Immune Cell Infiltration and Single-Cell Analysis**

IOBR is an immune-oncology computational tool utilized for studies in tumor immunobiology^25^. Here, based on expression profiles, we employed the xCell method within IOBR to calculate immune infiltration cell scores for 64 different immune cell types in each sample ^26^. The results obtained from xCell were visualized using the “corplot” and “ggplot2” packages in R. Subsequently, we conducted correlation analysis using the Spearman rank correlation test between immune cells and hub DEGs.

The Human Protein Atlas database (HPA, https://www.proteinatlas.org/) is a systematic human proteomics project aimed at providing comprehensive data on human protein expression, including protein expression profiles in different tissues and cell types, as well as subcellular localization information. This study utilized the HPA database to explore single-cell analysis data associated with feature gene.

**Statistical Analysis and Visualization**

The statistical analyses involved in this study were conducted using GraphPad Prism 9.0 and SPSS 27.0. The Shapiro-Wilk test was employed to assess the assumption of normal distribution. For data that conformed to normal distribution and passed the test for homogeneity of variances, the t-test method was applied, with significance set at p < 0.05. All data are presented as mean ± SD. Figure S1 was adapted from Servier Medical Art (http://smart.servier.com/), a reputable source recognized for its high-quality medical illustrations. Under a Creative Commons Attribution 3.0 Generic License (https://creativecommons.org/licenses/by/3.0/), the materials from Servier Medical Art permit their unrestricted distribution and modification, provided that appropriate credit is attributed.

**References**

1. Quenby S, Gallos ID, Dhillon-Smith RK, et al. Miscarriage matters: the epidemiological, physical, psychological, and economic costs of early pregnancy loss. *Lancet.* 2021;397(10285):1658-1667.

2. Wilcox AJ, Weinberg CR, O'Connor JF, et al. Incidence of early loss of pregnancy. *N Engl J Med.* 1988;319(4):189-194.

3. Zinaman MJ, Clegg ED, Brown CC, O'Connor J, Selevan SG. Estimates of human fertility and pregnancy loss. *Fertil Steril.* 1996;65(3):503-509.

4. ACOG Practice Bulletin No. 200: Early Pregnancy Loss. *Obstet Gynecol.* 2018;132(5):e197-e207.

5. Al Naimi A, Moore P, Brüggmann D, Krysa L, Louwen F, Bahlmann F. Ectopic pregnancy: a single-center experience over ten years. *Reprod Biol Endocrinol.* 2021;19(1):79.

6. Shen YT, Yang YY, Zhang PG, et al. Tubal ectopic pregnancy: a retrospective cohort study on clinical characteristics, treatment options and reproductive outcomes within 5 years. *Arch Gynecol Obstet.* 2022;306(6):2055-2062.

7. Zhang D, Shi W, Li C, et al. Risk factors for recurrent ectopic pregnancy: a case-control study. *Bjog.* 2016;123 Suppl 3:82-89.

8. Hennessy M, Dennehy R, Meaney S, et al. Clinical practice guidelines for recurrent miscarriage in high-income countries: a systematic review. *Reprod Biomed Online.* 2021;42(6):1146-1171.

9. du Fossé NA, van der Hoorn MP, van Lith JMM, le Cessie S, Lashley E. Advanced paternal age is associated with an increased risk of spontaneous miscarriage: a systematic review and meta-analysis. *Hum Reprod Update.* 2020;26(5):650-669.

10. Mann LM, Kreisel K, Llata E, Hong J, Torrone EA. Trends in Ectopic Pregnancy Diagnoses in United States Emergency Departments, 2006-2013. *Matern Child Health J.* 2020;24(2):213-221.

11. Bouyer J, Coste J, Shojaei T, et al. Risk factors for ectopic pregnancy: a comprehensive analysis based on a large case-control, population-based study in France. *Am J Epidemiol.* 2003;157(3):185-194.

12. Ticconi C, Capogna MV, Martelli F, et al. Ectopic pregnancy in women with recurrent miscarriage. *J Obstet Gynaecol Res.* 2018;44(5):852-860.

13. Kolben TM, Rogatsch E, Vattai A, et al. PPARγ Expression Is Diminished in Macrophages of Recurrent Miscarriage Placentas. *Int J Mol Sci.* 2018;19(7).

14. Panelli DM, Phillips CH, Brady PC. Incidence, diagnosis and management of tubal and nontubal ectopic pregnancies: a review. *Fertil Res Pract.* 2015;1:15.

15. Buck Louis GM, Sapra KJ, Schisterman EF, et al. Lifestyle and pregnancy loss in a contemporary cohort of women recruited before conception: The LIFE Study. *Fertil Steril.* 2016;106(1):180-188.

16. Tur-Torres MH, Garrido-Gimenez C, Alijotas-Reig J. Genetics of recurrent miscarriage and fetal loss. *Best Pract Res Clin Obstet Gynaecol.* 2017;42:11-25.

17. Colley E, Hamilton S, Smith P, Morgan NV, Coomarasamy A, Allen S. Potential genetic causes of miscarriage in euploid pregnancies: a systematic review. *Hum Reprod Update.* 2019;25(4):452-472.

18. Dinc K, Issın G. Novel marker to predict rupture risk in tubal ectopic pregnancies: the systemic immune-inflammation index. *Ginekol Pol.* 2023;94(4):320-325.

19. Wang X, Huang L, Yu Y, Xu S, Lai Y, Zeng W. Risk factors and clinical characteristics of recurrent ectopic pregnancy: A case-control study. *J Obstet Gynaecol Res.* 2020;46(7):1098-1103.

20. Guo Q, Li Z, Jia S, Tong F, Ma L. Mechanism of Human Tubal Ectopic Pregnancy Caused by Cigarette Smoking. *Reprod Sci.* 2023;30(4):1074-1081.

21. Diagnosis and Management of Ectopic Pregnancy: Green-top Guideline No. 21. *Bjog.* 2016;123(13):e15.

22. Franz M, Rodriguez H, Lopes C, et al. GeneMANIA update 2018. *Nucleic Acids Res.* 2018;46(W1):W60-w64.

23. Johnson WE, Li C, Rabinovic A. Adjusting batch effects in microarray expression data using empirical Bayes methods. *Biostatistics.* 2007;8(1):118-127.

24. Izmirlian G. Application of the random forest classification algorithm to a SELDI-TOF proteomics study in the setting of a cancer prevention trial. *Ann N Y Acad Sci.* 2004;1020:154-174.

25. Zeng D, Ye Z, Shen R, et al. IOBR: Multi-Omics Immuno-Oncology Biological Research to Decode Tumor Microenvironment and Signatures. *Front Immunol.* 2021;12:687975.

26. Aran D, Hu Z, Butte AJ. xCell: digitally portraying the tissue cellular heterogeneity landscape. *Genome Biol.* 2017;18(1):220.
