# Supplementary material for: Transcriptomic analysis reveals the landscape of the shared gene network between ectopic pregnancy and early pregnancy loss
Source: Genes Dis. 2025 Mar 27;12(6):101616. doi: 10.1016/j.gendis.2025.101616 (PMC12275975; doi:10.1016/j.gendis.2025.101616)
Supplement: Multimedia component 2 [file mmc2.docx]

**Supplementary Table 1 Clinical Characteristics of the women included in this study.**

|  | **Sequencing Group** | | | | **Validation Group** | | | |
| --- | --- | --- | --- | --- | --- | --- | --- | --- |
|  | NP (n=5) | EPL (n=5) | EP (n=5) | P value | NP (n=7) | EPL (n=7) | EP (n=7) | P value |
| **Age (year)** | 27.60 ± 1.82 | 28.20 ± 1.10 | 27.80 ± 1.30 | 0.801 | 27.43 ± 1.27 | 27.27 ± 1.27 | 28.00 ± 1.63 | 0.734 |
| **Gestational weeks (day)** | 57.20 ± 4.97 | 58.20 ± 3.35 | 58.80 ± 4.92 | 0.851 | 56.00 ± 3.70 | 56.43 ± 4.96 | 56.57 ± 3.65 | 0.965 |
| **BMI** | 21.53 ± 1.79 | 20.85 ± 0.90 | 21.07 ± 1.17 | 0.582 | 21.88 ± 0.80 | 21.41 ± 1.13 | 21.26 ± 1.58 | 0.620 |
| **Smoking status** | None | None | None | - | None | None | None | - |

EP: Ectopic pregnancy; EPL: Early pregnancy loss; NP: Control group.

**Supplementary Table 2 Quality assessment of raw data.**

| Sample | Read | Total reads | Total bases(bp) | GC% | Error rate (%) | Q20(%) | Q30(%) |
| --- | --- | --- | --- | --- | --- | --- | --- |
| Control_1 | Read_1 | 53,635,504 | 8,045,325,600 | 49.11 | 0.24 | 97.34 | 93.26 |
|  | Read_2 | 53,635,504 | 8,045,325,600 | 49.51 | 0.30 | 96.67 | 91.72 |
| Control_2 | Read_1 | 51,777,302 | 7,766,595,300 | 48.97 | 0.24 | 97.42 | 93.39 |
|  | Read_2 | 51,777,302 | 7,766,595,300 | 49.36 | 0.29 | 96.80 | 91.92 |
| Control_3 | Read_1 | 55,663,518 | 8,349,527,700 | 49.66 | 0.24 | 97.41 | 93.38 |
|  | Read_2 | 55,663,518 | 8,349,527,700 | 50.05 | 0.27 | 97.01 | 92.43 |
| Control_4 | Read_1 | 42,430,535 | 6,364,580,250 | 48.69 | 0.24 | 97.43 | 93.45 |
|  | Read_2 | 42,430,535 | 6,364,580,250 | 48.97 | 0.32 | 96.43 | 91.18 |
| Control_5 | Read_1 | 50,426,033 | 7,563,904,950 | 48.92 | 0.24 | 97.36 | 93.28 |
|  | Read_2 | 50,426,033 | 7,563,904,950 | 49.33 | 0.26 | 97.12 | 92.64 |
| EPL_1 | Read_1 | 53,832,765 | 8,074,914,750 | 49.08 | 0.26 | 97.20 | 93.00 |
|  | Read_2 | 53,832,765 | 8,074,914,750 | 49.39 | 0.30 | 96.64 | 91.83 |
| EPL_2 | Read_1 | 49,166,082 | 7,374,912,300 | 49.30 | 0.24 | 97.45 | 93.42 |
|  | Read_2 | 49,166,082 | 7,374,912,300 | 49.80 | 0.25 | 97.32 | 93.00 |
| EPL_3 | Read_1 | 49,134,503 | 7,370,175,450 | 46.98 | 0.25 | 97.31 | 93.16 |
|  | Read_2 | 49,134,503 | 7,370,175,450 | 47.40 | 0.27 | 96.96 | 92.32 |
| EPL_4 | Read_1 | 49,336,965 | 7,400,544,750 | 48.32 | 0.24 | 97.40 | 93.35 |
|  | Read_2 | 49,336,965 | 7,400,544,750 | 48.62 | 0.27 | 96.99 | 92.35 |
| EPL_5 | Read_1 | 47,548,525 | 7,132,278,750 | 48.13 | 0.23 | 97.45 | 93.44 |
|  | Read_2 | 47,548,525 | 7,132,278,750 | 48.52 | 0.27 | 96.99 | 92.29 |
| EP_1 | Read_1 | 49,532,673 | 7,429,900,950 | 47.46 | 0.25 | 97.27 | 93.10 |
|  | Read_2 | 49,532,673 | 7,429,900,950 | 47.66 | 0.27 | 96.96 | 92.37 |
| EP_2 | Read_1 | 46,400,480 | 6,960,072,000 | 45.80 | 0.24 | 97.38 | 93.21 |
|  | Read_2 | 46,400,480 | 6,960,072,000 | 46.00 | 0.28 | 96.93 | 92.24 |
| EP_3 | Read_1 | 44,103,527 | 6,615,529,050 | 47.08 | 0.24 | 97.41 | 93.41 |
|  | Read_2 | 44,103,527 | 6,615,529,050 | 47.35 | 0.34 | 96.21 | 90.63 |
| EP_4 | Read_1 | 42,200,443 | 6,330,066,450 | 47.36 | 0.24 | 97.43 | 93.40 |
|  | Read_2 | 42,200,443 | 6,330,066,450 | 47.72 | 0.31 | 96.53 | 91.31 |
| EP_5 | Read_1 | 47,907,845 | 7,186,176,750 | 48.14 | 0.23 | 97.47 | 93.52 |
|  | Read_2 | 47,907,845 | 7,186,176,750 | 48.45 | 0.33 | 96.34 | 90.87 |

GC%: The percentage of guanine (G) and cytosine (C) bases in the sequenced DNA; Q20(%): The percentage of bases in the sequencing data with a quality score of 20 or higher; Q30(%): The percentage of bases in the sequencing data with a quality score of 30 or higher.

**Supplementary Table 3** **The top 10 shared DEGs in 12 algorithnms.**

| MCC | DMNC | MNC | Degree | EPC | BottleNeck | EcCentricity | Closeness | Radiality | Betweenness | Stress | ClusteringCo-efficient |
| --- | --- | --- | --- | --- | --- | --- | --- | --- | --- | --- | --- |
| PTPRC | CCL3 | TYROBP | TYROBP | ITGB2 | PTPRC | PARVG | PTPRC | PTPRC | PTPRC | PTPRC | TMC8 |
| ITGB2 | FOLR2 | PTPRC | PTPRC | PTPRC | TLR4 | DOCK2 | TYROBP | TYROBP | TYROBP | TYROBP | STK32B |
| CD86 | CD200R1 | ITGB2 | ITGB2 | TYROBP | TYROBP | WAS | ITGB2 | ITGB2 | TLR4 | TLR4 | ARHGEF6 |
| TLR2 | LGALS9 | FCER1G | FCER1G | FCER1G | SYK | RGS18 | FCER1G | FCER1G | ITGB2 | ITGB2 | KLHL6 |
| TLR4 | ARHGAP25 | TLR4 | TLR4 | CD86 | SPP1 | PIK3CG | TLR4 | TLR4 | FCER1G | FCER1G | PRG4 |
| FCGR2A | WDFY4 | CD86 | CD86 | CYBB | TREM2 | HAVCR2 | CD86 | CYBB | PLEK | C1QA | DOK3 |
| TYROBP | MRC1 | LCP2 | LCP2 | TLR4 | WAS | SYK | CYBB | LCP2 | LCP2 | LCP2 | CCL4L2 |
| CCL2 | FCGR2A | CYBB | CYBB | CD68 | MAFB | PLAT | LCP2 | CD86 | CCL2 | CCL2 | RNASE1 |
| CCL3 | TLR6 | TLR2 | TLR2 | CTSS | LCP2 | CCL2 | CCL2 | CCL2 | C1QA | PLEK | GIMAP1-GIMAP5 |
| CTSS | AIF1 | CCL2 | CCL2 | TLR2 | C1QA | APOB | TLR2 | TLR2 | RUNX3 | CYBB | GIMAP2 |

**Supplementary Table 4 Top 10 candidate drug predicted using DSigDB.**

| Drug name | -Log_10_(adjusted P-value) | Odds Ratio | Combined Score | Gene |
| --- | --- | --- | --- | --- |
| Cholecalciferol CTD 00005655 | 6.76 | 12.32 | 267.35 | LRRC25, CD300A, ITGB2, F13A1, PLD4, NR4A2, MS4A6A, CYP24A1, RGS1, SPP1, CD14, TLR4, TLR2 |
| Pergolide HL60 UP | 9.13 | 7.48 | 210.20 | CD86, SASH3, FCER1G, GPR65, RNASE6, FPR1, SLA, CYBB, ARRB2, RUNX3, SAMSN1, ARHGAP25, NR4A2, HCK, TYROBP, MAFB, PTPRC, CYTH4, FCGR2A, LPAR6, AXL, RGS1, CASP1, SIGLEC7 |
| Beclometasone HL60 UP | 3.37 | 16.25 | 198.68 | MAFB, ADORA3, FPR1, SLA, VSIG4, SAMSN1 |
| Mebendazole HL60 UP | 9.13 | 7.01 | 195.15 | CD86, SASH3, MCTP1, FPR1, SLA, ARRB2, SAMSN1, GNA15, CYTH4, RGS1, C3AR1, SPP1, CCL2, CD14, FCER1G, CD300A, RUNX3, NR4A2, SLC7A7, MAFB, PTPRC, FCGR2A, AXL, LCP2, SIGLEC7 |
| Sodium dichromate CTD 00000827 | 7.32 | 8.31 | 193.53 | LST1, NCF4, RNASE6, LY96, RNASE1, AIF1, LAT2, SLC7A7, ADORA3, SLCO2B1, STAB1, TBXAS1, CCL3, CCL2, VSIG4, CD14, DOCK2, LAIR1 |
| Tesmilifene CTD 00001953 | 1.99 | 24.89 | 191.73 | CD86, PTPRC, CD14 |
| ACMC-20mvek CTD 00002629 | 5.06 | 10.57 | 181.73 | CD86, CYP27A1, MSR1, EDN1, SLCO2B1, ITGB2, CCL2, CD14, APOB, TLR4, TLR2 |
| Rimexolone HL60 UP | 2.28 | 17.53 | 154.15 | MAFB, BIN1, ADORA3, FPR1 |
| Fenoterol HL60 UP | 4.01 | 10.18 | 143.27 | CD86, CYTH4, PTPRC, FCGR2A, MAFB, RGS1, AXL, LPAR6, SLA |
| 1,3-Dimethylthiourea CTD 00001818 | 1.77 | 19.15 | 135.11 | MSR1, EDN1, CCL2 |

**Supplementary Table 5 Drug and protein structure data included in this study.**

| **Receptor** | **PBD ID** | **Ligand** | **Pubchem ID** |
| --- | --- | --- | --- |
| ITGB2 | 7USM | Cholecalciferol | 5280795 |
| TLR4 | 4G8A | Cholecalciferol | 5280795 |
| TLR2 | 2Z80 | Cholecalciferol | 5280795 |

**Supplementary Table 6 Primer sequences for qRT-PCR.**

| **Gene** | **Primer** | |
| --- | --- | --- |
| *β-actin* | F-Primer | TGGCACCCAGCACAATGAA |
|  | R-primer | CTAAGTCATAGTCCGCCTAGAAGCA |
| *CYBB* | F-Primer | ATTCTGGTTTGGCTGGGGTT |
|  | R-primer | CCAGTGCTGACCCAAGAAGTT |
