# Supplementary material for: Transcriptomic analysis reveals the landscape of the shared gene network between ectopic pregnancy and early pregnancy loss
Source: Genes Dis. 2025 Mar 27;12(6):101616. doi: 10.1016/j.gendis.2025.101616 (PMC12275975; doi:10.1016/j.gendis.2025.101616)
Supplement: Multimedia component 3 [file mmc3.pdf]

Transcriptomic Analysis Reveals the Landscape of the Shared Gene Network between Ectopic Pregnancy and Early Pregnancy Loss

Mengyu Jing[a,b,c,d1], Ying Zhou[a,b,c,d1], Shuyue Zheng[a,b,c,d], Yahui Xie[a,b,c,d], Xi Chen[a,b,c,d], Aixia Liu[a,b,c,d*]

[a]Department of Reproductive Endocrinology, Women's Hospital, Zhejiang University School of Medicine, 1 Xueshi Road, Hangzhou 310006, P.R. China.

[b]Key Laboratory of Reproductive Genetics (Ministry of Education), Zhejiang University, Hangzhou 310006, P.R. China.

[c]Zhejiang Key Laboratory of Maternal and Infant Health, Zhejiang University, Hangzhou 310006, P.R. China.

[d]Zhejiang Provincial Clinical Research Center for Child Health, Women's Hospital, Zhejiang University School of Medicine, Hangzhou, Zhejiang, 310006, P.R. China.

[*]Corresponding Authors: Aixia Liu, liuaixia@zju.edu.cn.

This PDF file includes:

Figure S1-S6

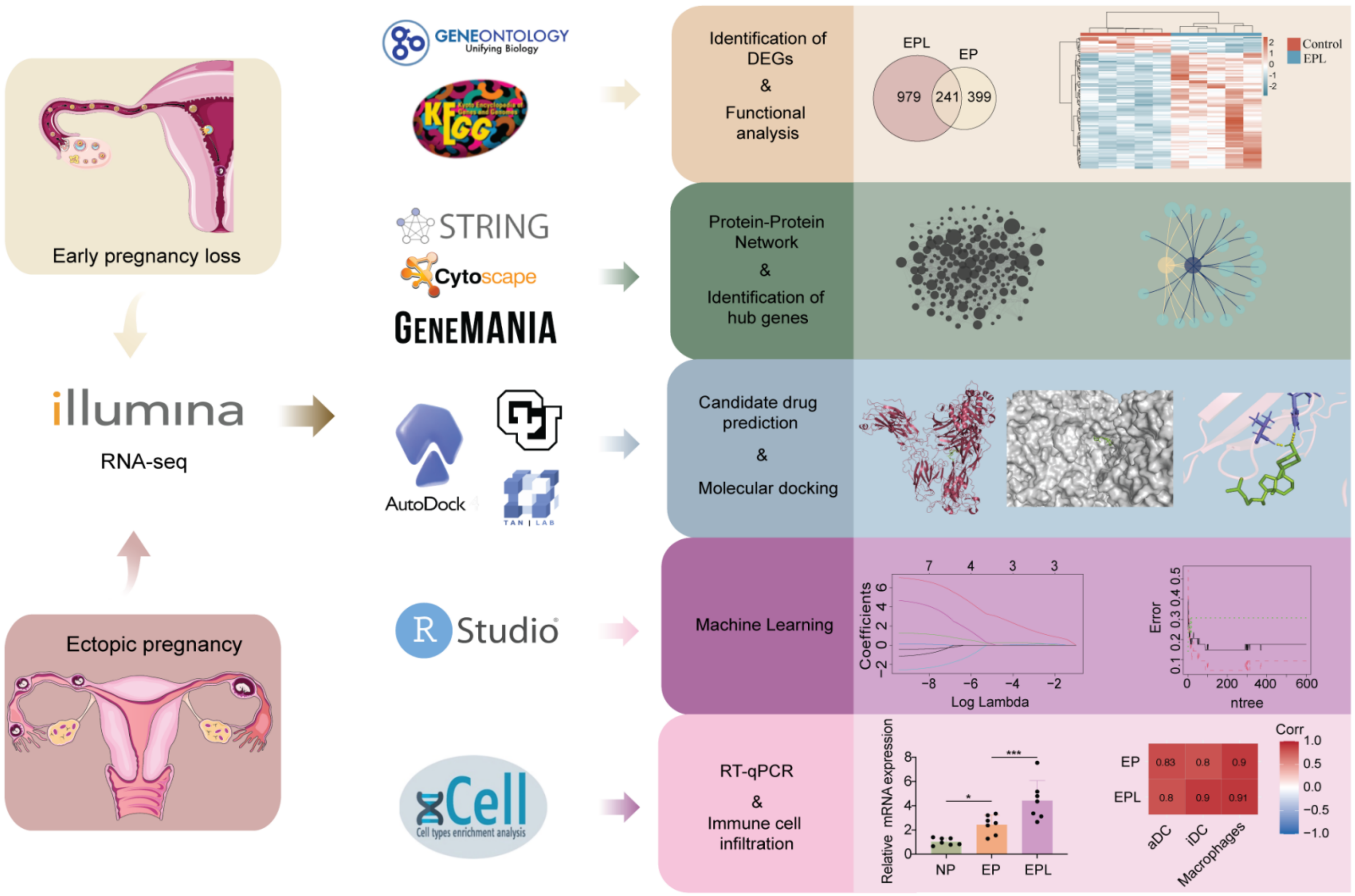

Fig. S1 The research workflow. This workflow diagram illustrates the analytical pipeline employed in this study to investigate early pregnancy loss and ectopic pregnancy, highlighting the integration of gene expression profiling, bioinformatics tools, machine learning, and experimental validation methods.

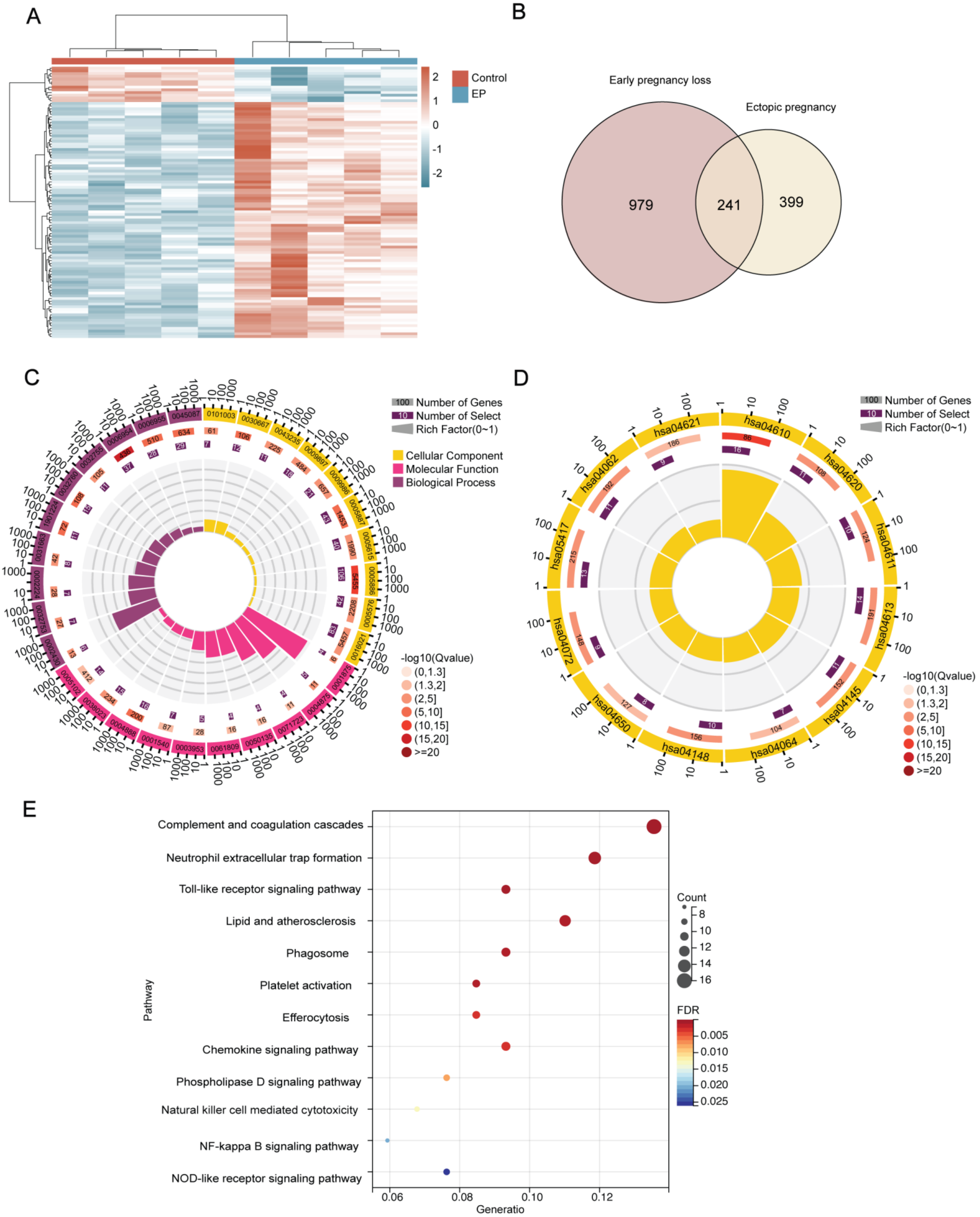

Fig. S2 The altered gene expression profiles and functional enrichment analysis of shared DEGs. (A) The heatmap of differentially expressed genes (DEGs) in EP patients. (B) The shared DEGs in EPL/EP patients. (C) The circular plot of GO analysis for shared DEGs. (D-E) KEGG analysis for shared DEGs. EP: Ectopic pregnancy; EPL: Early pregnancy loss.

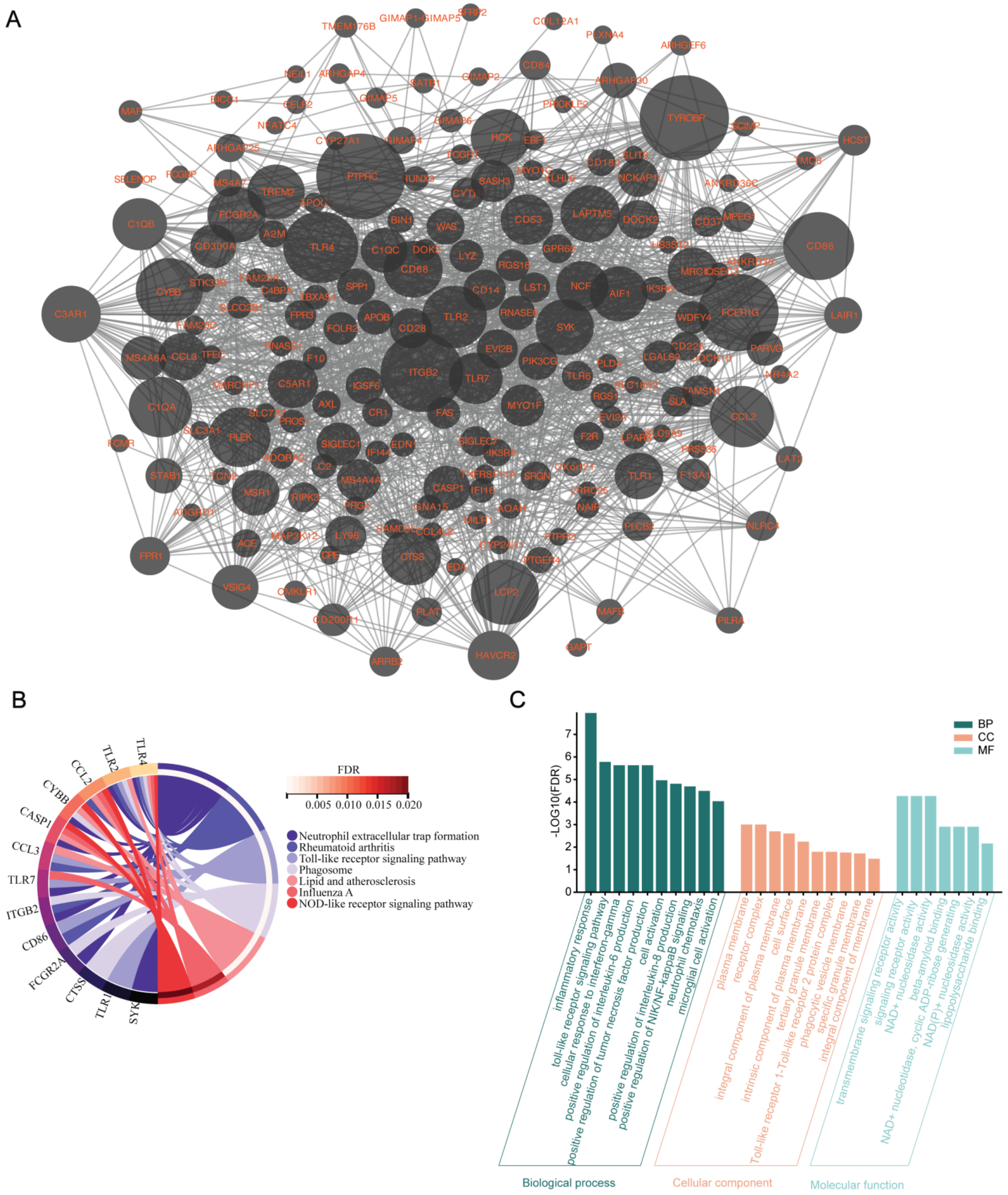

Fig. S3 PPI network analysis of shared DEGs. (A) The Protein-Protein Interaction network of shared DEGs. (B) The KEGG analysis of core module. (C) The GO analysis of core module. BP: Biological process; CC: Cellular component; MF: Molecular function.

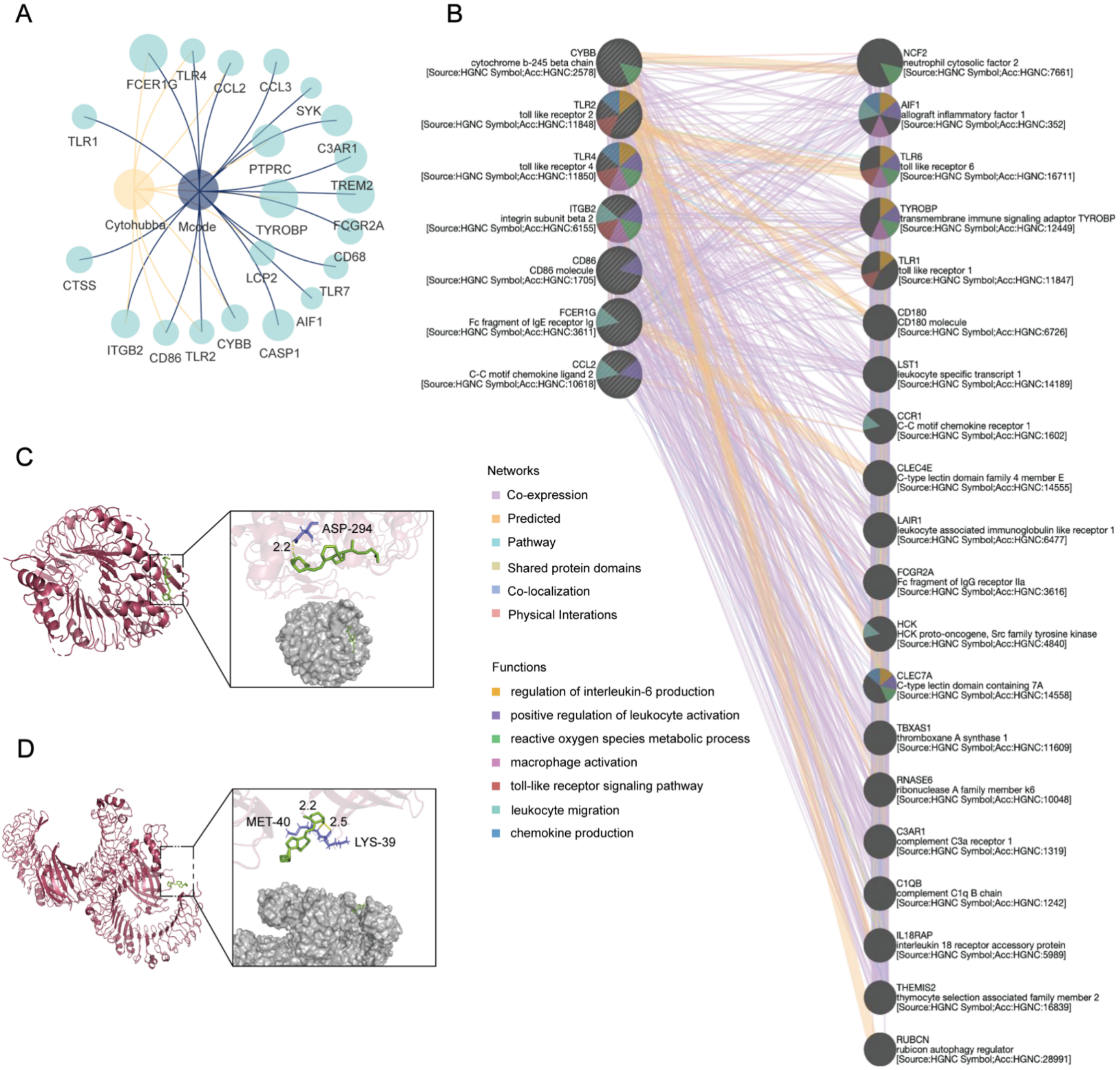

Fig. S4 Identification of feature DEGs and molecular docking of predicted drugs. (A) The overlap of DEGs between MCODE and CytoHubba. (B) Functional analysis of hub genes using GeneMANIA. (C) Molecular docking schematic of cholecalciferol and *TLR2*. (D) Molecular docking schematic of cholecalciferol and *TLR4*.

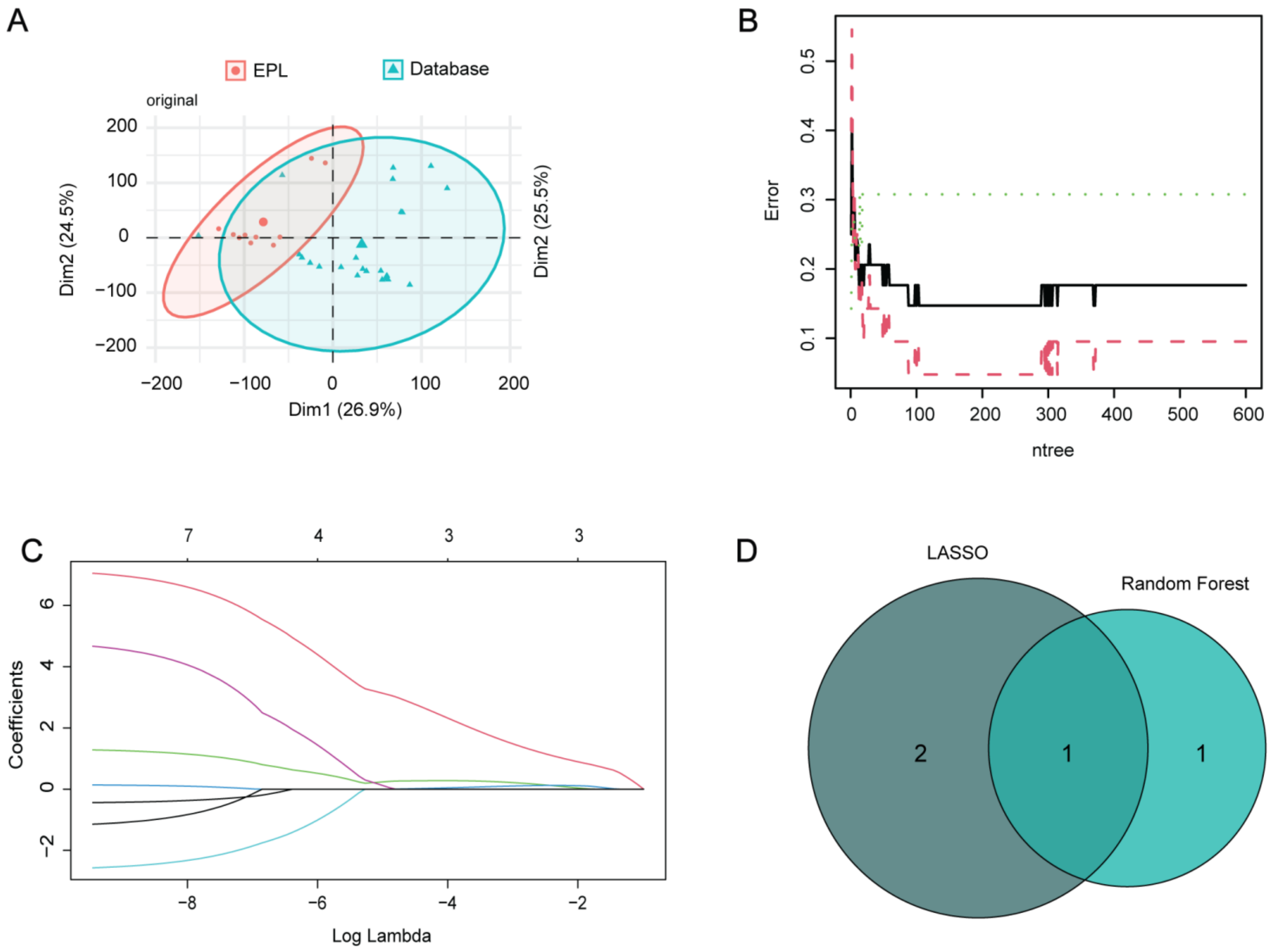

Fig. S5 Identification of signature hub-shared DEGs in EPL/EP patients. (A) PCA plots before batch correction with dataset GSE123719. (B) Machine learning of Random Forest analysis. (C) Machine learning of LASSO analysis. (D) Overlap of gene from machine learning. EPL: Early pregnancy loss; EP: Ectopic pregnancy.

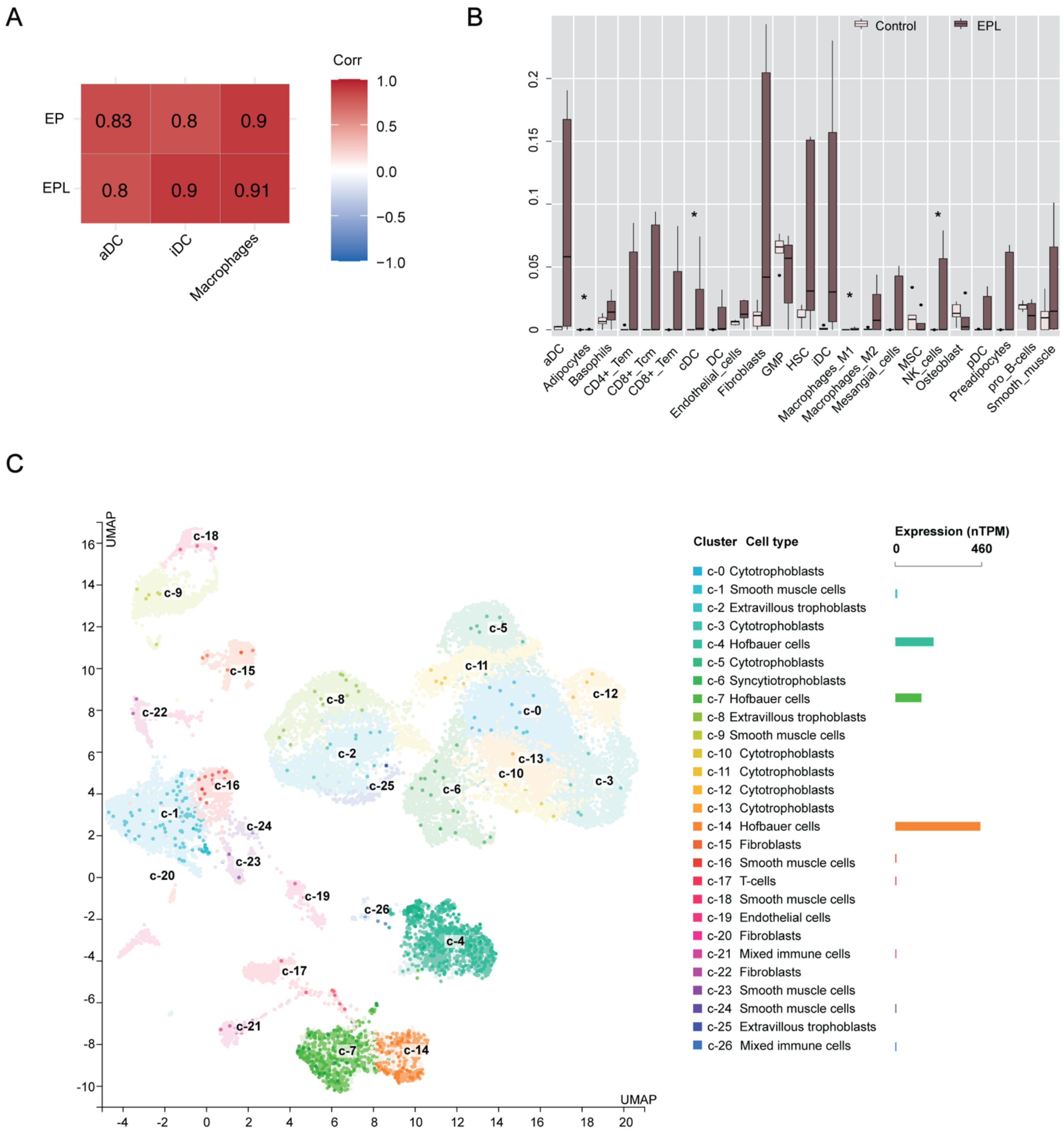

Fig. S6 Immune infiltration analysis in patients with EPL and EP. (A) Correlations between *CYBB* and immune cell components in EP/EPL patients. (B) Comparison of differentially expressed immune cell fractions between EPL patients and healthy controls (extremely low levels of immune cells are excluded). (C) Single-cell atlas of the *CYBB* gene in placental tissues. EPL: Early pregnancy loss; EP: Ectopic pregnancy; DC: Dendritic cell. *$p < 0.05$.
